# Cholesterol in the ciliary membrane as a therapeutic target against cancer

**DOI:** 10.3389/fmolb.2023.1160415

**Published:** 2023-03-16

**Authors:** Sotai Kimura, Tomoka Morita, Kosuke Hosoba, Hiroshi Itoh, Takashi Yamamoto, Tatsuo Miyamoto

**Affiliations:** ^1^ Department of Molecular Pathology, Graduate School of Medicine, Yamaguchi University, Ube, Japan; ^2^ Department of Molecular and Cellular Physiology, Graduate School of Medicine, Yamaguchi University, Ube, Japan; ^3^ Program of Biomedical Science, Graduate School of Integrated Sciences for Life, Hiroshima University, Hiroshima, Japan; ^4^ Program of Mathematical and Life Science, Graduate School of Integrated Sciences for Life, Hiroshima University, Hiroshima, Japan

**Keywords:** primary cilia, cholesterol, cancers, sonic hedgehog signal, Wnt signal

## Abstract

Primary cilium is a non-motile, antenna-like structure that develops in the quiescent G_0_ phase-cell surface. It is composed of an array of axonemal microtubules polymerized from the centrosome/basal body. The plasma membrane surrounding the primary cilium, which is called the ciliary membrane, contains a variety of receptors and ion channels, through which the cell receives extracellular chemical and physical stimuli to initiate signal transduction. In general, primary cilia disappear when cells receive the proliferative signals to re-enter the cell cycle. Primary cilia thus cannot be identified in many malignant and proliferative tumors. In contrast, some cancers, including basal cell carcinoma, medulloblastoma, gastrointestinal stromal tumor, and other malignancies, retain their primary cilia. Importantly, it has been reported that the primary cilia-mediated oncogenic signals of Hedgehog, Wnt, and Aurora kinase A are involved in the tumorigenesis and tumor progression of basal cell carcinoma and some types of medulloblastoma. It has also been demonstrated that cholesterol is significantly more enriched in the ciliary membrane than in the rest of the plasma membrane to ensure Sonic hedgehog signaling. A series of epidemiological studies on statin drugs (cholesterol-lowering medication) demonstrated that they prevent recurrence in a wide range of cancers. Taken together, ciliary cholesterol could be a potential therapeutic target in primary cilia-dependent progressive cancers.

## 1 Introduction

Primary cilium is a single, non-motile, antenna-like structure on the quiescent G_0_ phase-cell surface. The plasma membrane surrounding the primary cilium is composed of axonemal microtubules that originate from the centrosome-converted basal body, and is known as the ciliary membrane (Singla and Reiter, 2006). The ciliary membrane contains a variety of receptors and ion channels, thereby receiving the extracellular mechanical and chemical stimuli to transduce the signals involved in cell proliferation and differentiation (Fabbri et al., 2019).

In general, primary cilia are assembled in the quiescent G_0_ phase and disassembled in the cell cycle after receiving proliferative stimuli (Fabbri et al., 2019). It has been reported that primary cilia are not formed in many types of malignant tumors to dampen cilia-related signal transduction (Seeley et al., 2009; Hassounah et al., 2013; Nobutani et al., 2014; Liu et al., 2019). However, certain types of medulloblastoma, gastrointestinal stromal tumor, and basal cell carcinoma retain the primary cilia, which transduce signals such as Hedgehog and Wnt to contribute to the malignancy (Hassounah et al., 2012; Castiella et al., 2013; Park et al., 2021; Marino, 2022; Youn et al., 2022). Thus, whether primary cilia promote or suppress the progression of carcinoma is an important but difficult question in the field of oncology.

**FIGURE 1:**
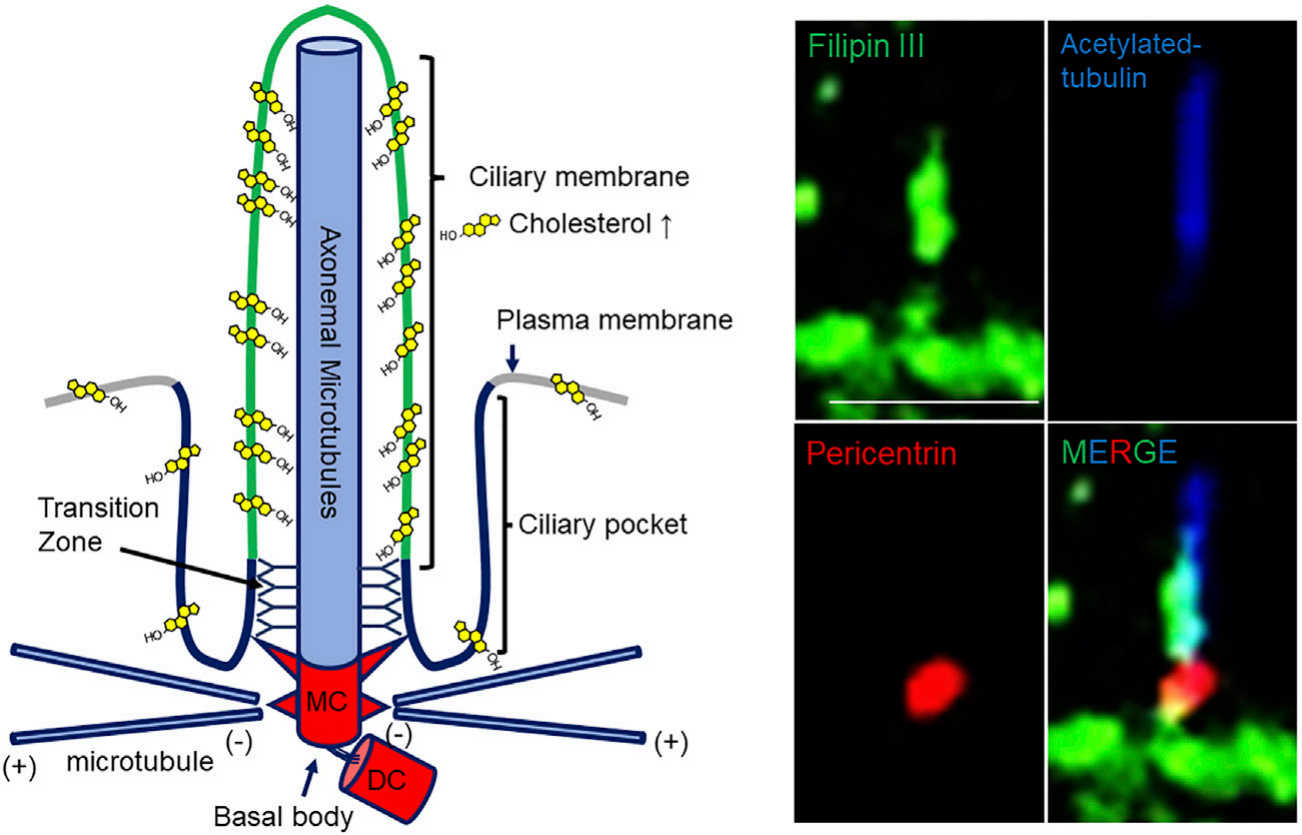
Schematic longitudinal section and cholesterol imaging of a primary cilium. (Left) The mother central body (MC) and daughter central bodies (DC) of the basal body (centrosome) are located at the base of the primary cilium, and microtubular axonemes develop from the mother central body docked to the cell membrane. The ciliary membrane covering the axonemes is rich in cholesterol and sphingolipids. At the base of the primary cilia, the plasma membrane is concave, forming a serial pocket. At the transition zone (TZ), the cytoplasm and ciliary membrane of primary cilia are biochemically compartmentalized from other cytoplasmic and cell membranes. (Right) Imaging of ciliary cholesterol in quiescent G_0_-phase hTERT-RPE1 cells stained with Filipin III. They were also immunostained with anti**-**acetylated-tubulin and anti-pericentrin antibodies. The merged image indicates the enriched ciliary cholesterol. Scale bar, 5 μm.

For primary cilia to receive extracellular signals effectively, not only the protein, but also the lipid composition of the ciliary membrane is compartmentalized from the rest of the plasma membrane (Nishimura et al., 2021; Miyamoto et al., 2022). Cholesterol, which is a major lipid for controlling cell membrane fluidity and is used as a precursor of steroid hormones and bile acids, is enriched in the ciliary membrane to transmit cilia-related oncogenic sonic hedgehog (SHH) signal (Miyamoto et al., 2022; Figure 1). Notably, epidemiological and experimental studies have suggested that cholesterol-lowering drug statins, which strongly inhibit the *de novo* cholesterol synthesis-limiting enzyme, hydroxymethylglutaryl-CoA (HMG-CoA) reductase, significantly prevent the recurrence in several cancers (Beckwitt et al., 2018; Riscal et al., 2019; Yang et al., 2020). These findings suggest that downregulation of ciliary cholesterol acts as tumor suppression. In this minireview, we summarize the function of primary cilia in the context of cancer formation and progression, and the potential of ciliary cholesterol as a therapeutic target.

## 2 Primary cilia-related signal transduction in the context of tumorigenesis

Primary cilia mediate cancer-related signaling such as the Sonic hedgehog, Wingless/INT (Wnt), NOTCH, platelet-derived growth factor (PDGF), mammalian target of rapamycin (mTOR), and Hippo pathways (Basten and Giles, 2013; Liu et al., 2018; Fabbri et al., 2019). Here, we focus on two major cilia-related cancer signal pathways.

### 2.1 SHH signaling pathway

**FIGURE 2:**
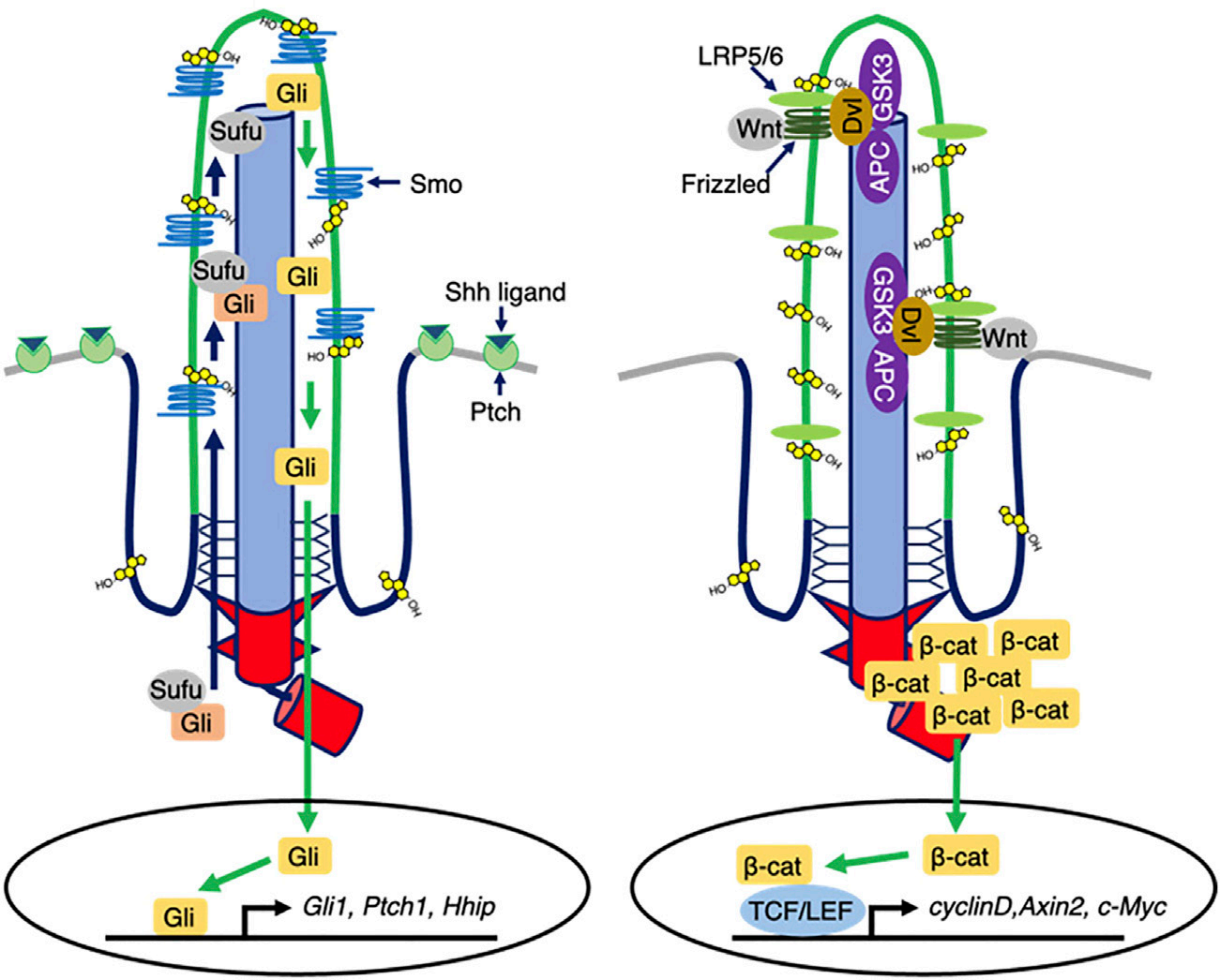
Ciliary cholesterol-mediated signal transduction. (Left) In the presence of Shh ligands, Ptch exits from ciliary membrane, while Smo enters and is then activated. Ciliary Smo allows Sufu to release Gli. Activated Gli is translocated into the nucleus, thereby controlling the transcription of Shh responsive genes. Ciliary cholesterol directly binds to Smo to ensure the activation. (Right) In the presence of Wnt1 class ligands, Frizzled-LRP5/6 activates DVL that destabilizes the destruction complex composed of APC and GSK3, thereby allowing β-catenin cytosolic accumulation and nuclear translocation to regulate the Wnt responsive gene expression.

SHH signaling is involved in the dorsal-ventral axis and in mid-line formation, digit patterning, and cerebellum development in embryogenesis (Harfe et al., 2004; Ribes and Briscoe, 2009; Wang et al., 2022). When the SHH ligand binds to the PTCH1 receptor, which is the 12-transmembrane patched 1 protein localized on the ciliary membrane in the absence of SHH ligand, it translocates to the outside of the cilia. Following the ciliary exit of PTCH1, the seven-transmembrane protein smoothened (SMO) enters the ciliary membrane to induce the release of GLI transcriptional protein known as glioma-associated oncogene from the Suppressor of fused (*SUFU*) protein in the ciliary cytoplasm, thereby shuttling GLI from the primary cilium compartment to the nucleus to control the transcription of GLI-target genes (Figure 2; Basten and Giles, 2013; Byrne et al., 2016; Qi et al., 2018; Liu et al., 2018; Fabbri et al., 2019; Niewiadomski et al., 2019). Germline mutations of the *PTCH1* gene causes autosomal-dominant Gorlin syndrome with basal cell carcinoma and medulloblastoma predisposition. In Gorlin syndrome with dominant negative mutations of *PTCH1*, constitutive activation of SMO triggers the misregulation of SHH-mediated gene expression to contribute to the cancer disposition (Athar et al., 2014).

### 2.2 Wnt signaling pathway

Wnt signaling controls many biological events, including embryogenesis, stem cell maintenance, and tumorigenesis (Wang et al., 2012). In the canonical Wnt signaling pathway, the Wnt1 class ligands (Wnt2, Wnt3, Wnt3a, and Wnt8) bind the membrane protein complex of Frizzled (FZ) family proteins and lipoprotein receptor-related protein (LRP) 5/6 to form the FZ, LRP5/6, and cytoplasmic Dishevelled (DVL) protein complex and trigger the downstream cascade. Importantly, FZ and LRP5/6 localize to the ciliary membrane, implying that primary cilia might sense the Wnt signals. In the absence of canonical Wnt ligands, β-catenin is phosphorylated and ubiquitinated by the destruction complex (DC), which consists of adenomatous polyposis coli (APC), the scaffolding protein Axin, glycogen synthase kinase (GSK)-3β, and other regulators to be degraded (Haseeb et al., 2019; He et al., 2015; Phesse et al., 2016; Chae and Bothwell, 2018; Aros et al., 2021). The canonical Wnt-activated DVL inhibits the DC to stabilize β-catenin, thereby allowing β-catenin nuclear translocation to induce target gene expression for cell proliferation and cell differentiation (Figure 2; He et al., 2015; Phesse et al., 2016; Chae and Bothwell, 2018; Aros et al., 2021).

In the non-classical Wnt signaling pathway, the Wnt5a type ligands (Wnt4, Wnt5a, Wnt5b, Wnt6, Wnt7a, and Wnt11) trigger Wnt/planar cell polarity (PCP) or the Wnt/Ca^2+^ pathways for cell polarization and cell migration. Both pathways are initiated by binding of the Wnt5a-type ligands to FZ/retinoic acid-related orphan receptor (ROR)/tyrosine-protein kinase RYK. The Wnt/Ca^2+^ pathway is mediated by phospholipase C (PLC) and protein kinase C (PKC) to increase the intracellular Ca^2+^concentration, thereby activating the calcium/calmodulin-dependent protein kinase II (CAMKII) and nuclear factor of activated T-cells (NFAT) pathways to regulate target gene transcription. In the Wnt/PCP pathway, the binding of a Wnt ligand to FZ, ROR, and RYK receptors involves DVL-mediated small GTPase RHOA and Rac1 for the control of actin dynamics and Jun N terminal kinase (JNK) activation in the context of cell polarization and cell migration (Haseeb et al., 2019; Chae and Bothwell, 2018). Notably, the interaction of Wnt5a and FZ stimulates casein kinase 1 (CK1) to phosphorylate DVL2 at the centrosome, thereby forming DVL2 and a mitotic kinase polo-like kinase 1 (PLK1) complex. The DVL2–PLK1 complex activates human enhancer of filamentation 1 (HEF1) and Aurora Kinase A (AurA) to disassemble primary cilia (Pugacheva et al., 2007). HEF1/AurA phosphorylates histone deacetylase (HDAC) 6 to deacetylate not only histone but also tubulin, thereby inhibiting axonemal tubulin polymerization. Interestingly, the HEF1/AurA-HDAC6-mediated primary cilia disassembly axis is also activated by platelet-derived growth factor signaling. Together, primary cilia mediate the Wnt signals, while their formation is controlled by the Wnt signals (Pugacheva et al., 2007; Liu et al., 2018; Lee, 2020).

## 3 Ciliogenesis in the cancer tissues

Consistent with the basic relationship between cell cycle and primary cilia formation, it has been reported that primary cilia formation is disrupted in many cancer tissues and cell lines. Heterochronic activation of mitotic kinases at centrosomes during the G_0_/G_1_ phases occurs in many cancers. AurA, Nemo-like kinase 2 (NEK2), and PLK1 phosphorylate HDAC6, a mother centriole capping protein, KIF24, and a microtubule-depolymerizing enzyme, KIF2A, respectively, to activate the primary cilia resorption program in many cancers (Kinzel et al., 2010; Izawa et al., 2015; Kim et al., 2015; Miyamoto et al., 2015). In small intestinal and colorectal cancer, the frequency of primary cilia was very low (median, 0.49%) in the cells of small bowel adenocarcinoma and 0.22% (median) in the cells of colorectal adenocarcinoma. Overall survival of the patients with a higher frequency of primary cilia (≥0.187%) was significantly longer (Dvorak et al., 2016). However, several papers demonstrated that primary cilia exist even in cancer tissues. In 25% (25/100) of pancreatic adenocarcinoma cases, most primary cilia were found in the lumen of well-differentiated adenocarcinomas. Primary cilia formation was also reported in 100% (8/8) of adenocarcinoma of the small intestine cases, 100% (32/32) of adenocarcinoma of the colorectum cases, and 72% (69/96) of clear cell renal cell carcinoma of the kidney cases (Emoto et al., 2014; Dvorak et al., 2016; Rozsypalova et al., 2019). In thyroid cancer, primary cilia are found in papillary carcinoma and follicular carcinoma; however, primary cilia are negative in Hürthle cell carcinoma, oncocytic subtype of papillary carcinoma, and papillary carcinoma associated with Hashimoto thyroiditis, which are often associated with abnormal mitochondrial function (Lee et al., 2021). These findings suggest that primary cilia in heterogenous cancer cell populations contribute to the oncogenic characteristics.

Recently, epithelial–mesenchymal transition transcriptional factors (SNAIL, SLUG, and ZEB1) induce primary cilia formation in mammary tumor-initiating cells of claudin-low breast cancers. These cilia receive FGFR1 signals to decrease the amount of GLIS2 protein at the cilia base for maintaining cancer stemness (Guen et al., 2017; Wilson et al., 2021). Medulloblastoma, a well-known cilia-related cancer, is an embryonal malignant tumor that predominantly affects children and is now histopathologically classified into five major types: Wnt-activated; SHH-activated and *TP53*-mutated; SHH-activated and *TP53* wild type; non-Wnt/non-SHH type; and histologically defined type, which contains classic medulloblastoma, desmoplastic/nodular medulloblastoma, medulloblastoma with extensive nodularity, and large cell/anaplastic medulloblastoma (WHO Classification of Tumours Editorial Board, 2021). The first two have primary cilia and show activation of the Wnt and SHH pathways, respectively (Marino, 2022; Youn et al., 2022). In medulloblastoma-modeled mice driven by the constitutively active *Smo* transgene (SmoM2), depletion of *Kif3a* removes primary cilia to block medulloblastoma formation (Barakat et al., 2013). In other cilia-related tumors, skin basal cell carcinoma and rhabdomyosarcoma often have primary cilia to mediate the constitutive activation of SHH signaling as a major driver of pathogenesis (Fu et al., 2014; Kuonen et al., 2019). The SMO antagonists, vismodegib and sonidegib, have been approved by the U.S. FOOD & DRUG administration (FDA) for treating skin basal cell carcinoma (Meiss and Zeiser, 2014; Jain et al., 2017). Together, primary cilia in several cancers transmit the oncogenic signals involved in tumorigenesis and progression.

## 4 Ciliary cholesterol as a therapeutic target in cancers

Structural biology studies of SMO have demonstrated that the extracellular cysteine rich domain (CRD) and the transmembrane domain (TMD) of Smo directly binds to cholesterol to activate SHH signaling (Nedelcu et al., 2013; Huang et al., 2018; Deshpande et al., 2019). In the absence of SHH ligand, PTCH1 can bind and efflux cholesterol from cells, thereby restricting cholesterol supply to Smo (Bidet et al., 2011). When SHH ligand binds to PTCH1 to inhibit its cholesterol efflux activity, a specific fraction of cholesterol in the plasma membrane called accessible cholesterol increases to bind to the CRD and TMD of Smo, thereby activating the smo and its downstream molecules (Kinnebrew et al., 2019). Other fraction of cholesterol termed sequestered cholesterol consists of cholesterol bound to phospholipids such as sphingomyelin (SM). Interestingly, depletion of SM by a antibiotics myricion, which inhibits the enzyme of SM synthesis, increases the amount of accessible cholesterol to enhance the Smo activation (Kinnebrew et al., 2019). Depletion of cellular cholesterol with methyl-β-cyclodextrin inhibits SHH signaling, suggesting that lowering the level of cholesterol promotes anti-cancer effects. Indeed, it was reported that statins reduce cancer recurrence and mortality by prohibiting the outgrowth of dormant cancer cells (Beckwitt et al., 2018). In breast cancer, estrogen receptor (ER)-negative breast cancer is particularly correlated with statin sensitivity, and fluvastatin has been clinically used to enhance tumor cell apoptosis in ER-negative breast cancer (Garwood et al., 2010). Clinical trials of combined treatment of standard anti-cancer drugs and statins are also underway in various other cancer types including head and neck, pulmonary, gastric, colorectal, hepatic, pancreatic, renal, prostatic, endometrial, and ovarian cancers, malignant lymphoma and multiple myeloma; and glioblastoma (Longo et al., 2020; Jiang et al., 2021). Because of the side effects of statins, which include rhabdomyolysis and liver damage in some cases, more specific cell membrane cholesterol-lowering technology could provide safer cancer therapies.

Ciliary cholesterol is a potential target for inhibiting oncogenic signaling. Our previous report demonstrated that peroxisomes move along the microtubules to supply cholesterol to the ciliary membrane, and that peroxisome biogenesis disorder Zellweger syndrome patient cells show defects in ciliary cholesterol levels and the SHH signal response. In the context of peroxisome-mediated ciliary cholesterol trafficking, small G protein Rab10, its GTP exchange factor Rabin8, and a kinesin molecule KIFC3 that moves to the minus end of microtubules, form a complex on the peroxisomal membrane (Miyamoto et al., 2020). Inhibitors of peroxisome dynamics might act more specifically to lower ciliary cholesterol. Mass spectrometry of the membranes of isolated cilia from sea urchin embryos revealed that several oxysterols synthesized by HSD11β2 at the endoplasmic reticulum are enriched to stimulate SMO (Raleigh et al., 2018). Importantly, genetic ablation of *HSD11B2* reduced the size of medulloblastoma in the SmoM transgenic mouse model, suggesting that HSD11β2 inhibitors might be applied as cilia-related cancer therapies (Daggubati et al., 2021).

Whether ciliary cholesterol regulates other oncogenic signalings is an important open question. WNT signal is a strong candidate of ciliary cholesterol-mediated oncogenic signal. Recently, it was reported that the WNT co-receptor LRP6 localizes to ciliary membrane to meditate the signal (Zhang et al., 2023). Cholesterol in the plasma membrane is enriched around the LRP6 and its binding partner Fz. WNT receptors and Cholesterol complex ensures the membrane recruitment and activation of DVL for the WNT/β-catenin signal transduction (Sheng et al., 2014). These findings suggest that ciliary membrane mediates the WNT/β-catenin signal for the proliferation of cancer cells and maintenance of cancer stem cells. Taken together, further investigation of ciliary cholesterol levels will enable exploration of new drugs against cancer.

## 5 Conclusion

In general, primary cilia have been considered to be absent in malignant and proliferative tumors. However, morphological observation of a wide variety of cancer tissues has revealed that there are carcinomas with primary cilia, which contribute to tumor formation and progression and the maintenance of cancer stemness through cilia-related signaling. Therefore, it is suggested that ciliary signal inhibition may be a therapeutic target and that statin therapy may be effective in inhibiting tumor progression. Ciliary cholesterol is involved in SHH signaling in skin basal cell carcinoma and medulloblastoma, while further investigations are needed to clarify which cilia-related signaling pathways are dependent on ciliary cholesterol for the application of statins and ciliary cholesterol agents as cancer therapeutics.
